# Consumption of ultra-processed foods is associated with cognitive status in elderly patients

**DOI:** 10.3389/fnut.2026.1839722

**Published:** 2026-06-02

**Authors:** Margherita Grasso, Francesca L’Episcopo, Marco Antonio Olvera-Moreira, Giuseppe Toscano, Stefano Muratore, Maria Angela Tripodi, Sabrina Musso, Veronica Bentivegna, Lucrezia Costanzo, Giusi Fatati, Melannie Toral-Noristz, Raynier Zambrano-Villacres, Lisandra León Brizuela, Raffaele Ferri, Giuseppe Lanza, Filippo Caraci

**Affiliations:** 1 Oasi Research Institute-IRCCS, Troina, Italy; 2 Carrera de Medicina, Universidad Católica de Santiago de Guayaquil, Guayaquil, Ecuador; 3 Escuela de Medicina, Universidad Espíritu Santo, Samborondón, Ecuador; 4 Facultad de Ciencias de la Salud y Desarrollo Humano, Universidad ECOTEC, Samborondon, Ecuador; 5 Universidad Internacional Iberoamericana, Campeche, Mexico; 6 Universidade Internacional do Cuanza, Cuito, Bié, Angola; 7 Fundación Universitaria Internacional de Colombia, Bogotá, Colombia; 8 Department of Surgery and Medical-Surgical Specialties, University of Catania, Catania, Italy; 9 Department of Drug and Health Sciences, University of Catania, Catania, Italy

**Keywords:** food processing, inflammation, mild cognitive impairment, neuroinflammation, ultra-processed foods

## Abstract

**Background:**

Emerging evidence suggests that there might be an association between excess consumption of ultra-processed foods (UPFs) on cognitive health. UPF intake could promote systemic inflammation, oxidative stress phenomena, and metabolic dysregulation, contributing to neurodegeneration onset and cognitive decline in elderly population.

**Aim:**

The aim of this cross-sectional study was to examine the relation between UPF dietary pattern on MCI status in elderly patients taking into account the contribution of inflammatory markers.

**Design:**

The dietary intake was assessed using a validated food frequency questionnaire in ninety-two participants. All reported food items were categorized according to the NOVA system, classifying foods on the basis of the extent and purpose of industrial processing. Plasmatic concentrations of TGF-β1 and TNF-ɑ were measured by ELISA assay at the time of baseline neuropsychological evaluation. The Mini-Mental State Examination (MMSE) and the Montreal Cognitive Assessment (MoCA) were administered to evaluate the cognitive function in all participants. Non-parametric tests, correlation analysis, and logistic regression models were performed to assess the relations between variables of interest.

**Results:**

No significant associations were observed for unprocessed/minimally processed foods, culinary processed foods, or processed foods across the different regression models. In contrast, higher consumption of UPF was associated with increased odds of MCI (adjusted OR = 4.24, 95% CI: 1.05–17.13). However, after additional adjustment for inflammatory biomarkers (TGF-*β* and TNF-*α*), the association was attenuated and no longer statistically significant (OR = 4.79, 95% CI: 0.73–31.24), although the direction of the association remained positive.

**Conclusion:**

UPF consumption may be associated with increased likelihood of MCI, and inflammatory status may potentially play a role in this association.

## 1 Introduction

Cognitive impairment and dementia represent major and growing public health challenges worldwide (1). As populations age, the prevalence of mild cognitive impairment (MCI) and related neurodegenerative disorders continues to rise, leading to substantial individual, social, and economic burdens (2, 3). Although age and genetic susceptibility are important non-modifiable risk factors, an increasing body of evidence suggests that lifestyle and environmental exposures play a critical role in shaping cognitive trajectories across the lifespan (4). Among these, diet has emerged as a potentially modifiable determinant of brain health (5). In particular, nutritious and minimally processed diets, like the Mediterranean diet, have been consistently associated with lower risk of cognitive disorders (6), acting via various pathways, including modulating inflammatory response and gut microbiome (7).

In recent decades, profound changes in food systems and dietary patterns have led to a marked increase in the consumption of ultra-processed foods (UPFs), with nutritional, environmental and economic factors influencing their level of consumption differential among various populations (8–10). These products are typically industrial formulations characterized by high energy density, poor nutritional quality, and the presence of additives. It has been consistently reported that nowadays, UPFs account for a substantial proportion of daily caloric intake in many countries (11). High consumption of UPFs has been consistently associated with increased risk of obesity, cardiovascular disease, type 2 diabetes, and certain types of cancer (12, 13), contrary to traditional diets, like Mediterranean diet (14), characterized by high consumption of minimally processed foods and home-cooked dishes (15). More recently, attention has turned to their potential impact on neurological and cognitive outcomes, yet this area remains comparatively underexplored.

Several biological mechanisms have been proposed to explain how diets rich in ultra-processed foods could adversely affect cognitive health (16). Such products are often high in refined sugars, saturated fats, and sodium, while being low in fiber, vitamins, and bioactive compounds essential for optimal brain function (11, 17). In addition, UPFs may promote systemic inflammation, oxidative stress, and metabolic dysregulation, which are pathways increasingly recognized as central contributors to neurodegeneration and cognitive decline (16). Chronic low-grade inflammation, in particular, has been implicated in the pathophysiology of MCI and dementia, raising the possibility that inflammatory processes may mediate the relationship between poor dietary quality and impaired cognition (18).

Despite these plausible mechanisms, epidemiological evidence directly linking ultra-processed food consumption to cognitive impairment remains limited, and few studies have investigated the biological pathways underlying this association (19). Understanding whether inflammation contributes to the relationship between ultra-processed diets and cognitive health could provide valuable insights into potential targets for prevention and intervention.

The present study aimed to examine the association between UPF intake and MCI in a sample of adults referring to a geriatric unit due to subjective cognitive concerns. Additionally, the role of inflammation through examination of biomarkers was tested in order to explore their potential relation with the outcome of interest.

## 2 Methods

### 2.1 Study design and population

This study is based on a cross-sectional analysis of consecutive patients enrolled in the Geriatrics Unit of the IRCCS Oasi Research Institute-IRCCS in Troina (Italy). Inclusion criteria included: (i) diagnosis of subjective memory impairment, very mild cognitive decline, isolated memory deficit, mild cognitive decline, or mild vascular cognitive decline; (ii) age over 65 years. Exclusion Criteria included: (i) presence of other types of dementia or a diagnosis of dementia within the context of other neurological pathologies; (ii) diagnosis of major psychiatric disorders (major depression and psychosis). The Department for Cerebral Involution (Psychology Unit) adhering to the currently accepted criteria from the Diagnostic and Statistical Manual of Mental Disorders, Fifth Edition (DSM-5), assessed patients’ diagnosis. Out of 1,000 patients undergoing a visit for subjective cognitive (memory) concerns, 908 did not meet inclusion criteria (because diagnosed or suspected diagnosis of other pathological conditions) leaving 92 potential candidates to participate in the present study. All agreed to participate in the study (Supplementary Figure S1). The present study was conducted according to the Declaration of Helsinki, the Guidelines for Good Clinical Practice and its protocol was approved by the Ethics Committee of the Oasi Research Institute-IRCCS in Troina (Italy) (CEL-IRCCS OASI/10-04-2025/04; and Verbale n. 9 del 19/10/2023, Codice Locale Progetto: T5-AN-11). All participants provided written informed consent prior to participation.

### 2.2 Background characteristics

Information on sociodemographic characteristics, lifestyle behaviors, and health conditions was obtained through structured questionnaires and clinical examinations. Covariates considered in the analyses included age, sex, education level, body mass index (BMI), and smoking status. These variables were selected *a priori* based on previous literature and their potential role as confounders of the association between diet and cognitive function.

### 2.3 Dietary assessment and definition of UPF

Dietary intake was assessed using a validated food frequency questionnaire (FFQ) (20) administered by trained interviewers. Dietary habits were assessed by asking participants to indicate how frequently they had consumed, on average, each item from a list of 110 commonly consumed foods and beverages over the previous 12 months. For every item, nine frequency categories were available, ranging from “never or almost never” to “four to five times per day,” in order to capture habitual intake with adequate precision. The questionnaire incorporated standard portion sizes, which were used to estimate the typical quantity consumed on each occasion. Reported frequencies were translated into mean daily intakes using established conversion procedures, allowing the estimation of average consumption in grams or milliliters per day for each food item. Estimates of energy intake were obtained by linking individual consumption data to the Italian Food Composition Tables developed by the Research Center for Food and Nutrition (CREA). For each participant, overall daily energy intake was calculated by multiplying the composition of each food by its corresponding estimated daily amount and then summing the values across all items.

All reported food items were categorized according to the NOVA system, which classifies foods on the basis of the extent and purpose of industrial processing. Specifically, items were assigned to four groups: (1) unprocessed or minimally processed foods, including products such as grains, meat, fish, milk, eggs, fruits, vegetables, and nuts; (2) processed culinary ingredients, such as sugar, vegetable oils, and butter; (3) processed foods, including items like packaged breads and cheeses; and (4) UPFs, comprising products such as confectionery, savory snacks, fast foods, and sugar-sweetened beverages. Most food items included in the FFQ were directly and clearly identified as home-made and/or industrially produced, hence easily recognizable as UPF or other NOVA group categories.

For analytical purposes, the average daily intake (expressed in grams per day) of foods belonging to NOVA groups was calculated for each participant. These values were used to determine the proportion of unprocessed/minimally processed foods and UPFs relative to total daily food consumption weight. The use of a weight-based ratio was chosen to capture the real proportion of ultra-processed products within the total diet, minimizing the influence of differences in caloric density and allowing a more direct comparison between individuals with different overall energy intakes. This approach is consistent with a substantial body of epidemiological literature, largely because many UPFs (such as artificially sweetened beverages, low-fat products, or reformulated “diet” items) contribute little or no energy despite being highly processed. An energy-based metric would therefore systematically underrepresent these items, potentially leading to misclassification of individuals with high exposure to industrial formulations but low caloric intake from such sources. In contrast, weight-based measures more accurately capture the physical presence and frequency of UPFs in the diet, aligning better with the conceptual framework of food processing classification systems. Additionally, the structure of FFQs supports the use of weight-based estimates. Portion sizes are predefined and standardized, enabling relatively consistent quantification across participants. Conversely, energy content is derived indirectly through food composition tables and assumptions about recipes or composite dishes, which introduces additional uncertainty. This is particularly relevant for FFQ items that aggregate multiple foods or preparation methods under a single entry, where caloric estimation may not accurately reflect the true composition consumed by participants. To define the main exposure variables, participants were dichotomized based on the median ratio of each NOVA group, with individuals classified as having either higher or lower consumption according to this median cut-off.

### 2.4 Cognitive assessment and diagnosis of MCI

Cognitive function was evaluated using a standardized neuropsychological assessment battery that included the Mini-Mental State Examination (MMSE) (21, 22) and the Montreal Cognitive Assessment (MoCA) (23). The Mini-Mental State Examination (MMSE) is a brief, clinician-administered instrument used to obtain a quantitative assessment of global cognitive functioning. It comprises 30 items that cover a range of cognitive abilities, including orientation to time and place (10 points), short-term and delayed recall (6 points), attention and calculation (5 points), language skills (8 points), and visuoconstructive abilities (1 point). The test involves a series of simple questions and tasks requiring verbal or written responses, which are scored as either correct or incorrect. Total scores can vary from 0 to 30, with higher values reflecting better cognitive performance. Standard interpretive guidelines were used, and scores falling below age- and education-adjusted normative thresholds were considered suggestive of cognitive impairment. The Montreal Cognitive Assessment (MoCA) is another 30-point screening tool, specifically developed to identify mild or early cognitive deficits. It includes 12 tasks assessing multiple domains: visuospatial and executive abilities (5 points), naming (3 points), attention and working memory (6 points), language (3 points), abstract reasoning (2 points), delayed memory recall (5 points), and orientation (6 points). The assessment incorporates a variety of response formats, such as multiple-choice items, drawing exercises, and brief verbal answers. In accordance with established recommendations, one additional point was added to the total score for participants with 12 or fewer years of education. Overall scores range from 0 to 30, with higher scores indicating better cognitive functioning; results below accepted normative cut-offs were interpreted as indicative of potential cognitive impairment. The MoCA was administered in order to enhance detection of subtle deficits, particularly in executive and attentional functions, which are commonly affected in the early phases of cognitive decline.

Mild cognitive impairment was defined according to established criteria, incorporating information on cognitive test performance, functional status, and absence of dementia. Participants were classified as having MCI if they met the following criteria: (i) subjective or informant-reported cognitive complaints, (ii) objective impairment in one or more cognitive domains based on age- and education-adjusted norms, (iii) largely preserved functional independence, and (iv) no diagnosis of dementia. Individuals not meeting these criteria were classified as cognitively normal. MCI was diagnosed according to the International criteria consistent with the Diagnostic and Statistical Manual of Mental Disorders, Fifth Edition (DSM-5). Specifically, patients with mainly single- or multiple domain memory deficits were defined “MCI amnesic” according to Petersen distinction, while MCI non-amnestic patients showed impairment in executive functions, language or visuospatial abilities (24). Total scores from MMSE and MoCa supported domain-specific deficits combined with the clinical evaluation and the functional assessment verified by modified barthel index (BADL) e IADL (instrumental activities of daily living).

### 2.5 Psychological covariates

To account for non-cognitive factors that could influence performance on neuropsychological tests, participants underwent assessment with established clinical screening measures. Symptoms of depression were evaluated using two complementary instruments. The Hamilton depression rating scale (HDRS) (25) is a clinician-administered questionnaire consisting of 17 primary items that examine core features of depressive disorders, including mood, feelings of guilt, sleep disturbances, anxiety, psychomotor changes, and somatic complaints. Items are rated on either 3- or 5-point scales, producing a total score between 0 and 52, with higher values reflecting more severe depressive symptoms. In parallel, the 15-item short form of the Geriatric Depression Scale (GDS-SF) (22), a self-report tool specifically designed for use in older adults, was also administered. The GDS-SF employs a simple yes/no response format and yields scores from 0 to 15, where higher scores indicate greater levels of self-reported depressive symptomatology. The use of both measures allowed for a comprehensive evaluation of mood, incorporating both clinician-rated and participant-reported perspectives. Excessive daytime sleepiness was assessed with the Epworth Sleepiness Scale (ESS) (26), an eight-item self-administered questionnaire that evaluates the likelihood of falling asleep in a variety of routine daily situations. Respondents rate each scenario on a 4-point scale ranging from 0 (“would never doze”) to 3 (“high chance of dozing”). The summed score ranges from 0 to 24, with higher totals indicating increased daytime somnolence and a greater probability of underlying sleep-related problems.

### 2.6 Assessment of inflammatory biomarkers

Blood samples were collected from participants at the time of the baseline neuropsychological assessment using standardized procedures consistent with those previously reported (27). Venous blood was drawn into EDTA-K2 anticoagulant tubes and processed shortly after collection. Samples were first centrifuged at 1900 rpm for 10 min to separate plasma from cellular components. The resulting plasma fraction underwent a second centrifugation step at 3900 rpm for 10 min to ensure complete removal of residual cells and debris. Following processing, plasma was divided into aliquots and stored at −80 °C until laboratory analyses were performed.

Plasma levels of transforming growth factor beta 1 (TGF-β1) and tumor necrosis factor alpha (TNF-*α*) were quantified using enzyme-linked immunosorbent assay (ELISA) techniques. Active human TGF-β1 concentrations were measured in plasma samples diluted 1:10, using a commercially available ELISA kit (Bio-Techne, R&D Systems; catalog DB100C) in accordance with the manufacturer’s instructions. TNF-*α* concentrations were similarly determined with a specific human TNF-*α* ELISA kit (Bio-Techne, R&D Systems; catalog DTA00D), following the protocol provided by the supplier. All samples were analyzed in duplicate, and the average of the two measurements was used. As specified by the manufacturer, the mean of minimum detectable dose (MDD) of human TGF-β1 was 2.38 pg./mL (ranged from 0.889–5.50 pg./mL) and we did not detect values below quantification limits and all measured values were above the LOD. The MDD of human TNF-*α* ranged from 2.09–6.23 pg./mL with a mean of 4.00 pg./mL, and for TNF-α measurement, all values of our samples were ranged or above the LOD.

Absorbance readings for each assay well were obtained with a Varioskan LUX Multimode Microplate Reader (Agilent BioTek, Santa Clara, CA, USA) at wavelengths of 450 nm, 540 nm, and 570 nm. To account for potential optical artifacts, background correction was performed by subtracting the absorbance values measured at 540 nm or 570 nm from those obtained at 450 nm, as recommended for ELISA data processing.

### 2.7 Statistical analysis

Participant characteristics were summarized across categories of UPF consumption using means and standard deviations for continuous variables and frequencies and percentages for categorical variables. Differences between groups were assessed using t-tests, ANOVA, or chi-square tests, respectively, as appropriate. Multivariable logistic regression models were used to estimate the association between UPF intake and odds of MCI. Results were expressed as odds ratios (ORs) and 95% confidence intervals (CIs). Sequential models included adjustment for total energy intake (model 1), age, sex, smoking status and educational level (model 2); and further adjustment for inflammatory biomarkers (model 3). Diagnostic checks were performed to assess the robustness of logistic regression models. Multicollinearity was evaluated using variance inflation factors (VIFs), influential observations were assessed through leverage values, standardized residuals, and Cook’s distance, and sparse data were examined by inspecting the distribution of events across exposure categories. The linearity assumption for continuous covariates was assessed using restricted cubic splines. Model calibration was evaluated by comparing observed and predicted probabilities. All statistical analyses were conducted using SPSS 29 (SPSS Inc., Chicago, IL, USA). A two-sided *p*-value < 0.05 was considered statistically significant.

## 3 Results

### 3.1 Study population and background characteristics

The final analytic sample comprised 92 participants, of whom 37 were diagnosed with MCI. Participants were stratified according to the level of UPF consumption into low (*n* = 69) and high (*n* = 23) intake groups.

Overall, 45.7% of participants were male and 54.3% were female. No significant differences in sex distribution were observed between UPF consumption groups (*p* = 0.468). Participants with high UPF consumption were significantly older compared with those with lower consumption (76.1 ± 6.3 vs. 72.2 ± 6.8 years, *p* = 0.017). Mean body mass index (BMI) did not differ significantly between groups (29.3 ± 4.8 vs. 28.1 ± 4.2 kg/m^2^, *p* = 0.348).

Educational level differed significantly according to UPF intake (*p* = 0.016). Individuals with high UPF consumption were more likely to have a low educational level (65.2%) compared with those with low UPF intake (33.3%), whereas a higher proportion of participants with low UPF consumption had medium or high education. Smoking status did not differ significantly between groups (*p* = 0.668) (see Table 1).

**Table 1:** Background characteristics by level of UPF consumption in the study sample.

	Ultra-processed foods			
	Total (*n* = 92)	Low (*n* = 69)	High (*n* = 23)	*p*-value
Sex, *n* (%)				0.468
Male	42 (45.7)	33 (47.8)	9 (39.1)	
Female	50 (54.3)	36 (52.2)	14 (60.9)	
Age, mean (SD)	73.2 (6.8)	72.2 (6.8)	76.1 (6.3)	0.017
BMI, mean (SD)	28.4 (4.3)	28.1 (4.2)	29.3 (4.8)	0.348
Educational level, *n* (%)				0.016
Low	38 (41.3)	23 (33.3)	15 (65.2)	
Medium	46 (50.0)	38 (55.1)	8 (34.8)	
High	8 (8.7)	8 (11.6)	0 (0.0)	
Smoking status, *n* (%)				0.668
Smoker	33 (37.1)	24 (35.8)	9 (40.9)	
Non-smoker	56 (62.9)	43 (64.2)	13 (59.1)	

### 3.2 Clinical characteristics by consumption of UPF

Clinical characteristics according to UPF consumption are presented in Table 2. Participants with higher UPF intake had significantly lower cognitive performance scores. Specifically, mean MMSE scores were lower in the high UPF group compared with the low UPF group (25.2 ± 3.4 vs. 27.2 ± 2.5, *p* = 0.003). Similarly, MoCA scores were significantly lower among individuals with higher UPF consumption (21.2 ± 5.0 vs. 23.9 ± 3.9, *p* = 0.011).

**Table 2:** Clinical characteristics by level of UPF consumption in the study sample.

	Ultra-processed foods			
	Total (*n* = 89)	Low (*n* = 66)	High (*n* = 23)	*p*-value
MMSE, mean (SD)	26.7 (2.9)	27.2 (2.5)	25.2 (3.4)	0.003
MoCa, mean (SD)	23.2 (4.3)	23.9 (3.9)	21.2 (5.0)	0.011
GDS, mean (SD)	3.1 (3.0)	2.8 (2.8)	4.0 (3.4)	0.104
HDRS, mean (SD)	5.3 (3.7)	5.0 (3.6)	6.3 (3.8)	0.165
ESS, mean (SD)	3.1 (2.7)	2.9 (2.8)	3.5 (2.5)	0.437

MMSE, mini-mental state examination; MoCa, montreal cognitive assessment, GDS, geriatric depression scale; HDRS, hamilton depression rating scale; ESS, Epworth sleepiness scale.

No statistically significant differences were observed between UPF groups for depressive symptoms or sleepiness measures. Mean GDS scores were slightly higher among high UPF consumers (4.0 ± 3.4 vs. 2.8 ± 2.8), but this difference did not reach statistical significance (*p* = 0.104). Likewise, HDRS scores (6.3 ± 3.8 vs. 5.0 ± 3.6, *p* = 0.165) and ESS scores (3.5 ± 2.5 vs. 2.9 ± 2.8, *p* = 0.437) did not differ significantly between groups.

### 3.3 Dietary intakes by consumption of UPF

Dietary intake by MCI status based on the NOVA classification is shown in Table 3. Overall, most unprocessed or minimally processed food groups did not differ significantly between participants with and without MCI.

**Table 3:** Mean weight ratio of major food groups according to the level of processing (based on NOVA classification) by MCI status in the study sample.

		MCI status		
		No (*n* = 55)*	Yes (*n* = 37)*	
	Mean g/day (SD)	Mean WR (SD)		*p*-value
Unprocessed/minimally processed foods				
Fruit	507.54 (450.24)	55.54 (44.67)	40.22 (41.38)	0.100
Vegetables	273.46 (291.44)	29.60 (30.70)	22.15 (24.16)	0.219
Legumes	29.48 (32.84)	2.97 (3.26)	2.71 (3.13)	0.702
Meat (red and white)	49.27 (27.53)	5.05 (2.90)	4.14 (2.30)	0.270
Fish and seafoods	42.24 (37.72)	3.98 (2.97)	4.30 (4.55)	0.678
Pasta or cereals	135.76 (96.43)	13.96 (9.82)	12.08 (8.70)	0.348
Eggs	1.79 (2.15)	0.17 (0.20)	0.18 (0.23)	0.828
Milk and unprocessed dairy	161.47 (143.47)	15.54 (11.73)	15.97 (16.91)	0.885
Nuts	17.98 (24.47)	2.10 (2.89)	1.23 (1.14)	0.084
Tea	55.73 (100.52)	5.97 (10.86)	4.60 (7.97)	0.512
Coffee	58.51 (37.24)	5.84 (3.71)	5.47 (3.53)	0.630
Butter	1.96 (4.12)	0.20 (0.47)	0.17 (0.27)	0.693
Sugar	1.60 (2.18)	0.17 (0.24)	0.14 (0.16)	0.509
Olive oil	9.27 (2.11)	0.89 (0.22)	0.91 (0.17)	0.643
Seed oils	1.60 (2.18)	0.17 (0.24)	0.14 (0.16)	0.509
100% fruit juice	45.1 (137.48)	6.68 (16.71)	0.98 (3.68)	0.044
Bread	188.86 (120.01)	18.43 (11.69)	18.28 (11.82)	0.953
Processed meat	7.57 (9.79)	0.80 (1.11)	0.64 (0.64)	0.432
Jam	3.49 (6.56)	0.35 (0.72)	0.33 (0.51)	0.913
Wine	65.64 (107.63)	6.48 (9.71)	6.25 (11.65)	0.921
Beer	13.81 (41.32)	1.77 (4.92)	0.70 (1.96)	0.212
Fast foods	0.52 (2.52)	0.07 (0.31)	0.02 (0.10)	0.388
Breakfast cereals	2.84 (7.92)	0.31 (0.80)	0.22 (0.72)	0.579
Carbonated sweetened beverages	23.22 (54.58)	2.69 (5.98)	1.63 (4.11)	0.351
Confectioned fruit juices	0.00 (0.00)	0.00 (0.00)	0.00 (0.00)	—
Ultraprocessed dairy foods	147.59 (148.68)	13.15 (11.77)	16.17 (16.13)	0.303
Sweets	56.59 (65.23)	5.44 (6.69)	5.60 (5.89)	0.906
Salty snacks	2.13 (13.16)	0.10 (0.28)	0.37 (2.00)	0.320
Liquors	1.79 (5.81)	0.27 (0.72)	0.03 (0.07)	0.051
Meat/milk analogues	7.34 (37.9)	0.74 (3.50)	0.68 (3.99)	0.938
Artificial sweeteners	0.00 (0.00)	0.00 (0.00)	0.00 (0.00)	—

Participants without MCI tended to consume higher amounts of fruits (55.54 ± 44.67 g/day vs. 40.22 ± 41.38 g/day), vegetables (29.60 ± 30.70 g/day vs. 22.15 ± 24.16 g/day), and nuts (2.10 ± 2.89 g/day vs. 1.23 ± 1.14 g/day), although these differences were not statistically significant.

Among all dietary components, only 100% fruit juice intake differed significantly between groups. Participants without MCI consumed higher amounts of 100% fruit juice compared with those with MCI (6.68 ± 16.71 g/day vs. 0.98 ± 3.68 g/day, *p* = 0.044). Consumption of other food categories, including bread, processed meat, sweets, salty snacks, and ultra-processed dairy products, did not differ significantly between groups.

### 3.4 Biomarkers of inflammation by consumption of UPF

**Figure 1:**
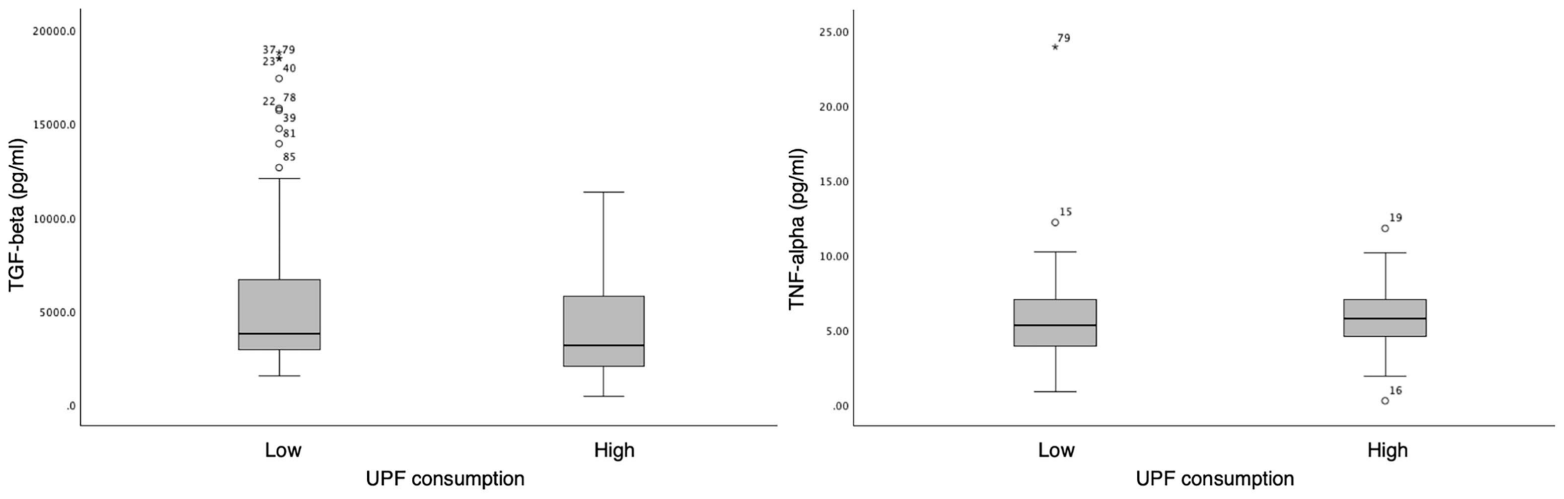
Mean TGF-beta (pg/ml) and TNF-alpha (pg/ml) levels by consumption of UPF.

Patients reporting higher consumption of UPF had lower levels of TGF-β1 (4300.4 ± 3162.4 pg./mL vs. 6032.9 ± 4735.0 pg./mL) and higher levels of TNF-*α* (6.01 ± 2.96 pg./mL vs. 5.87 ± 3.38 pg./mL) although the differences between groups were not statistically significant (*p* = 0.121 and *p* = 0.887, respectively; Figure 1).

### 3.5 Association between dietary intake by level of food processing and MCI status

The associations between food processing level and MCI status are presented in Table 4. Concerning logistic regression analyses, no evidence of problematic multicollinearity (all VIFs <1.2 and tolerance >0.890 for all continuous variables included in the most adjusted model), influential observations (standardized residuals, Cook’s distance, and leverage statistics), evidence of poor fit (Hosmer–Lemeshow goodness-of-fit, *p* > 0.05) major departures from linearity, or severe sparse-data issues was observed. However, some exposure categories contained limited numbers of events, which may have contributed to instability of estimates and wide confidence intervals.

**Table 4:** Association between foods according to the level of processing (based on NOVA classification) and MCI status.

	Likelihood of having MCI, OR (95% CI)	
	Low consumption	High consumption
Unprocessed/minimally processed foods		
Model 1^a^	1	0.51 (0.18–1.44)
Model 2^b^	1	1.04 (0.27–4.08)
Model 3^c^	1	2.23 (0.39–13.25)
Culinary processed foods		
Model 1^a^	1	0.53 (0.19–1.49)
Model 2^b^	1	0.48 (0.12–1.91)
Model 3^c^	1	0.23 (0.02–2.12)
Processed foods		
Model 1^a^	1	1.92 (0.74–5.02)
Model 2^b^	1	1.55 (0.41–5.86)
Model 3^c^	1	2.84 (0.42–19.37)
Ultra-processed foods		
Model 1^a^	1	5.19 (1.85–14.54)
Model 2^b^	1	4.24 (1.05–17.13)
Model 3^c^	1	3.36 (0.43–26.47)

a Adjusted for energy intake.

b Adjusted for age, sex, BMI, smoking status and educational level.

c Adjusted as model 2 + TGF-beta and TNF-alpha levels.

No significant associations were observed for unprocessed/minimally processed foods, culinary processed foods, or processed foods across the different regression models. In contrast, higher consumption of ultra-processed foods was associated with increased odds of MCI in partially adjusted models. In Model 1 (adjusted for energy intake), participants in the high UPF intake category had significantly greater odds of MCI compared with those in the low intake group (OR = 5.19, 95% CI: 1.85–14.54). After further adjustment for age, sex, smoking status, and educational level (Model 2), the association remained significant (OR = 4.24, 95% CI: 1.05–17.13).

However, after additional adjustment for inflammatory biomarkers (TGF-β1 and TNF-*α*) in Model 3, the association was attenuated and no longer statistically significant (OR = 4.79, 95% CI: 0.73–31.24), although the direction of the association remained positive, suggesting that higher consumption of ultra-processed foods may be involved in the MCI status through inflammatory biomarkers.

## 4 Discussion

In this cross-sectional study of elderly patients, higher consumption of UPFs was associated with greater likelihood of MCI. Participants in the highest category of UPF intake had significantly higher odds of MCI compared with those in the lowest category, independent of sociodemographic characteristics, lifestyle factors, and overall energy intake. Notably, the association considers the whole ultra-processed dietary pattern rather than individual foods, suggesting a role of diet quality in individuals consuming more UPFs compared to those consuming lower shares. However, adjustment for circulating biomarkers of inflammation substantially attenuated the magnitude of this association, suggesting that systemic inflammatory processes may play a role in the relationship between ultra-processed dietary patterns and cognitive health.

Although research on UPFs and brain health is relatively recent, our results are consistent with several large prospective investigations conducted in different populations (19). One of the earliest longitudinal analyses in this field was performed in the Brazilian Longitudinal Study of Adult Health (ELSA-Brasil), which followed more than 10,000 middle-aged adults for approximately 8 years (28). In that study, higher intake of UPFs was associated with significantly faster decline in global cognition and executive function over time (28). This longitudinal evidence supports the plausibility of our cross-sectional observations by demonstrating that diets rich in ultra-processed foods can precede and predict subsequent deterioration in cognitive performance. Further support comes from analyses of the UK Biobank, where investigators examined incident dementia outcomes in a large prospective cohort with more than 500 documented cases of dementia during a median 10-year follow-up (29). In that study, each 10% increase in the proportion of energy derived from ultra-processed foods was associated with a 25% higher risk of all-cause dementia, even after extensive adjustment for socioeconomic and lifestyle factors (29). Notably, substitution analyses suggested that replacing ultra-processed foods with minimally processed alternatives could meaningfully reduce dementia risk (29). These findings provide compelling evidence that the level of food processing itself, beyond traditional measures of diet quality, may be relevant for long-term cognitive health. More recently, the REasons for Geographic and Racial Differences in Stroke (REGARDS) project, a longitudinal study of Black and White adults in the United States evaluated the association between ultra-processed food intake and incident cognitive impairment among over 14,000 adults (30). The investigators found that higher UPF consumption was associated with a 16% increased risk of developing cognitive impairment per 10% increment in intake (30). Importantly, this association persisted after controlling for adherence to established healthy dietary patterns such as the Mediterranean, DASH, and MIND diets (30). These results reinforce the idea that food processing captures dimensions of diet not fully reflected by conventional nutrient-based or pattern-based indices, and that ultra-processed foods may exert specific adverse effects on the brain. Evidence from the Framingham Heart Study also points in the same direction. In this long-running US cohort, higher consumption of ultra-processed foods in midlife was associated with increased risk of Alzheimer’s disease over more than a decade of follow-up, particularly among individuals younger than 68 years at baseline (31). Participants consuming ten or more servings of UPFs per day had nearly threefold higher risk of developing Alzheimer’s disease compared with those with lower intake (31). Although the number of events was modest, this study highlights that exposure to highly processed diets earlier in adulthood may be especially relevant for later-life neurodegeneration (31). The overall body of evidence, summarized in a recent systematic review and meta-analysis of more than 800,000 individuals, indicates that high UPF consumption is associated with a substantially elevated risk of dementia across observational studies (19).

In this context, increasing scientific evidence hypothesized that dietary factors may target pathological processes involved in cognitive decline development such as neuroinflammation, impaired neurogenesis and synaptic plasticity, thus preventing or controlling its manifestation. Furthermore, it has been established that nutrition could modulate the immune system response and the neuroinflammatory processes involved in the pathogenesis of AD-related cognitive decline and in the neurodegenerative progression (32). Inflammation may affect cognitive health through multiple pathways, including endothelial dysfunction, reduced cerebral blood flow, disruption of the blood–brain barrier, and promotion of amyloid and tau pathology (33). Our finding showed that adjustment for inflammatory biomarkers weakened the association between UPF intake and MCI, consistently with this biological framework and supporting the hypothesis that inflammatory activation represents one pathway through which ultra-processed diets adversely influence the brain. It suggests that at least part of the detrimental cognitive effect of UPFs may operate through pathways involving immune activation and chronic inflammation. The recent evidence points to UPFs as negative contributors to inflammation with an association between higher inflammation scores and the average amount of UPF consumed daily (34, 35). UPFs and their individual components have been shown to be associated with intestinal and systemic inflammation, with a consequent reduction of anti-inflammatory cytokines and increased levels of pro-inflammatory cytokines and chemokines (36). Moreover, it has been demonstrated that the higher levels of UPF consumption are associated with inflammatory markers and are correlated with the worsening of depressive symptoms, known to be a risk factor for the development of AD-related cognitive decline (37, 38). Our data suggest that higher consumption of ultra-processed foods may be involved in the MCI status through the reduction of TGF-β1 and the increase of TNF-*α*.

Several mechanisms may explain how high consumption of UPFs could contribute to impaired cognitive function. Ultra-processed products are typically energy-dense and nutritionally poor, with high levels of added sugars, refined starches, saturated and trans fats, and sodium, combined with low amounts of fiber, vitamins, minerals, and phytochemicals (39). Such characteristics may promote metabolic dysregulation, oxidative stress, and alterations in the gut microbiome, all of which can stimulate chronic low-grade inflammation (40). On the contrary higher adherence to traditional diets, like Mediterranean diet, associated with higher probability of reaching the nutritional recommendations, being indicative of its richness in diverse nutrients (41), and being rich in both prebiotic and probiotics may mitigate inflammatory imbalance (42–44). UPFs are often characterised by an unfavorable nutrient composition, ultra-processed foods contain numerous additives, emulsifiers, artificial sweeteners, and compounds formed during industrial processing and packaging (45). Emerging experimental evidence suggests that some of these components may alter gut microbiota composition, increase intestinal permeability, and trigger immune activation (45). Chronic low-grade systemic inflammation resulting from these processes is increasingly recognized as a key contributor to neurodegeneration and vascular damage in the brain (45). This interpretation is coherent with extensive literature demonstrating that both dietary factors and inflammatory processes contribute to endothelial dysfunction, blood–brain barrier disruption, reduced cerebral perfusion, and neurodegenerative changes, all recognized drivers of cognitive impairment (46). Notably, it is also plausible that UPF consumption acts as a marker of broader unhealthy lifestyle patterns (47). Diets rich in UPFs are often accompanied by lower physical activity, poorer sleep quality, and higher prevalence of chronic diseases, all of which may independently contribute to cognitive impairment (47, 48). Although we adjusted for many of these factors, residual confounding cannot be entirely excluded.

The present study extends this evidence in several important ways. First, our analysis demonstrates that the association is already evident at earlier stages of cognitive dysfunction, namely MCI. Identifying modifiable dietary correlates of MCI is of particular relevance, as this stage represents a critical window for prevention and intervention. Second, few studies to date have explored biological pathways underlying the association between ultra-processed foods and cognitive health. Our observation that inflammatory biomarkers attenuate the association provides novel epidemiological support for inflammation as a potential mechanistic link. Moreover, this study has several strengths. The analysis was conducted in a well-characterized population with detailed information on diet, cognitive function, clinical characteristics, and possible new biomarkers. Dietary intake was assessed using a comprehensive and standardized instrument, and foods were classified according to the widely accepted NOVA system. Using a weight ratio also reduces potential bias related to the high energy density of many UPFs, enabling a more accurate assessment of dietary patterns based on the physical amount of foods consumed. Cognitive status was determined using validated neuropsychological assessments rather than self-report alone, enhancing the accuracy of outcome classification. The availability of objective inflammatory biomarkers allowed us to explore potential mechanisms underlying the observed associations.

Nevertheless, important limitations should be acknowledged. First, the cross-sectional design precludes causal inference and does not allow determination of the temporal sequence between diet and cognitive impairment. Reverse causation is possible, as individuals with early cognitive difficulties, especially older adults with MCI who may have altered food purchasing, meal preparation, or eating habits, may change their dietary habits or rely more heavily on convenient UPFs. Second, dietary intake was self-reported and therefore subject to recall or social desirability bias and measurement error. Also, the use of FFQs to address UPF consumption is a common limitation for epidemiological studies for several reasons. FFQs are not generally designed to identify and assess UPF consumption; the number of ultra-processed products is predefined depending on the original items included in the FFQ and may not necessarily reflect the totality of UPFs consumed, hence leading to an underestimation. However, this limitation represents a standardized error for the whole sample and a common limitation for all other existing studies sharing a similar methodology. Furthermore, the use of weight-based proportions also entails clear limitations. Because it gives equal importance to all grams consumed, it may overemphasize high-volume, low-energy UPFs (e.g., diet beverages or low-calorie products) while underrepresenting smaller but energy-dense items, potentially distorting the nutritional relevance of exposure. This can lead to attenuation or misinterpretation of associations if health outcomes are more strongly driven by caloric load or nutrient composition than by the sheer mass of UPFs consumed. However, the adjustment for energy intake in all logistic regression models should overcome such a limitation. Nonetheless, weight-based metrics are less directly comparable with dietary guidelines and metabolic endpoints that are inherently energy-based, complicating interpretation and cross-study comparisons. Third, biomarkers were measured at a single time point and may not fully capture long-term inflammatory status. Forth, the retrieved associations should be considered in light of the consideration that the relatively small sample of participants and cases may affect the statistical power of the analyses. Although we limited to the minimum the number of predictors reasonably needed to be considered for such type of analysis, we may not exclude that the number of events was still too small to fit all variables considered in the models. Nonetheless, although we adjusted for the most critical variables that may be reasonably considered confounders, residual confounding, such as socioeconomic factors, comorbidity burden, lifestyle or overall dietary patterns, may remain unmeasured. Finally, the generalizability of our findings may be limited to populations with similar demographic and dietary characteristics. The relatively small sample of elderly patients from a single Italian center limit the generalizability of the findings, but our results lay the basis for exploring the link between UPFs intake and cognitive functions in a community-based cohort.

In conclusion, our study suggests that higher consumption of UPFs may be associated with increased likelihood of MCI, and that this relationship may be partly explained by systemic inflammation. These findings add to growing concerns regarding the potential neurological consequences of highly processed dietary patterns and suggests inflammation as a plausible biological pathway. Given the rising global consumption of UPFs and the increasing burden of cognitive disorders, these results might have important public health implications. Dietary interventions aimed at reducing intake of ultra-processed products and promoting minimally processed, nutrient-rich foods may represent a promising strategy for preserving cognitive health. However, prospective studies with repeated dietary and biomarker measurements, as well as randomized interventions, are needed to confirm causality and to further elucidate underlying mechanisms.

## Data Availability

The data that support the findings of this study are available by the authors, upon reasonable request.
