# Supplementary material for: Consumption of ultra-processed foods is associated with cognitive status in elderly patients
Source: Front Nutr. 2026 Jun 2;13:1839722. doi: 10.3389/fnut.2026.1839722 (PMC13270669; doi:10.3389/fnut.2026.1839722)
Supplement: Supplementary file 1 [file Table_1.docx]

***Supplementary Material***

**Supplementary Figure 1.** Study design.


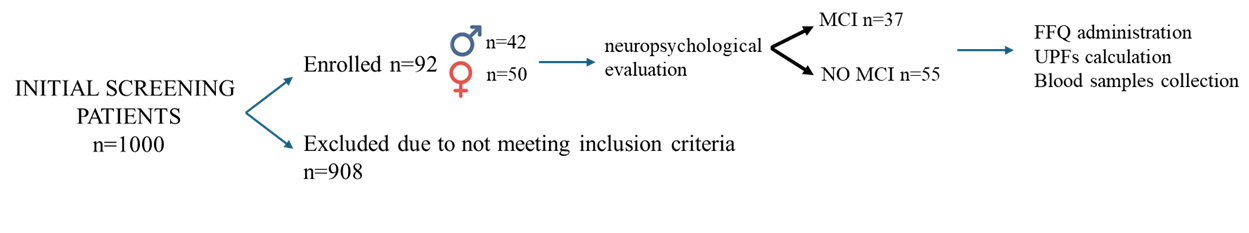


MCI, Mild Cognitive Impairment; FFQ, Food Frequency Questionnaire; UPF, ultra-processed foods
